# Exploring healthcare workers’ experiences of a simple intervention to reduce their intrusive memories of psychological trauma: an interpretative phenomenological analysis

**DOI:** 10.1080/20008066.2024.2328956

**Published:** 2024-03-27

**Authors:** Sara Ahmed Pihlgren, Lotta Johansson, Emily A. Holmes, Marie Kanstrup

**Affiliations:** a Department of Psychology, Uppsala University, Uppsala, Sweden; b Division of Psychology, Department of Clinical Neuroscience, Karolinska Institutet, Stockholm, Sweden; c The Neurosurgical Intensive Care Unit, Sahlgrenska University Hospital, Gothenburg, Sweden; d Institute of Health and Caring Sciences, The Sahlgrenska Academy, University of Gothenburg, Gothenburg, Sweden; e Department of Women's and Children's Health, Uppsala University, Uppsala, Sweden; f Theme Women’s Health and Allied Health Professionals, Medical Unit for Medical Psychology, Karolinska University Hospital, Solna, Sweden

**Keywords:** COVID-19, healthcare staff, digital intervention, intrusive memories, trauma, qualitative study, lived experiences, interpretative phenomenological analysis, COVID-19, personal sanitario, intervención digital, recuerdos intrusivos, trauma, estudio cualitativo, experiencias vividas, análisis fenomenológico interpretativo

## Abstract

**Background:** Many healthcare workers (HCWs) endured psychologically traumatic events at work during the coronavirus disease 2019 (COVID-19) pandemic. For some, these events are re-experienced as unwanted, recurrent, and distressing intrusive memories. Simple psychological support measures are needed to reduce such symptoms of post-traumatic stress in this population. A novel intervention to target intrusive memories, called an imagery-competing task intervention (ICTI), has been developed from the laboratory. The intervention includes a brief memory reminder cue, then a visuospatial task (Tetris® gameplay using mental rotation instructions for approximately 20 min) thought to interfere with the traumatic memory image and reduce its intrusiveness. The intervention has been adapted and evaluated in a randomized controlled trial (RCT) with Swedish HCWs (ClinicalTrials.gov identifier: NCT04460014).

**Objective:** We aimed to explore how HCWs who worked during the COVID-19 pandemic experienced the use of a brief intervention to reduce their intrusive memories of work-related trauma.

**Method:** Interpretative phenomenological analysis was used for in-depth understanding of the lived experiences of HCWs who used the intervention. Seven participants from the RCT were interviewed by an independent researcher without prior knowledge of the intervention. Interviews were conducted via telephone and transcribed verbatim.

**Results:** Four general themes were generated: ‘Triggers and troublesome images’, ‘Five Ws regarding support – what, when, why, by/with who, for whom’, ‘Receiving it, believing it, and doing it’ and ‘The intervention – a different kind of help’; the last two included two subthemes each. The results reflect participants’ similarities and differences in their lived experiences of intrusive memories, support measures, and intervention impressions and effects.

**Conclusion:** HCWs’ experiences of the novel ICTI reflect a promising appraisal of the intervention as a potential help measure for reducing intrusive memories after trauma, and gives us a detailed understanding of HCWs’ needs, with suggestions for its adaption for future implementation.

**Trial registration:** ClinicalTrials.gov identifier: NCT04460014.

## HIGHLIGHTS

- Many healthcare workers experience images or ‘flashbacks’ of traumatic experiences from their work during the COVID-19 pandemic.
- To ensure that individual needs are met, there is a need to tailor and refine current psychological support measures and their use for healthcare workers.
- The imagery-competing task intervention was perceived as acceptable, indicating its potential utility as a help measure to reduce intrusive memories after trauma.

## Abstract (es)

**Antecedentes:** Los trabajadores de la salud (HCW, por sus siglas en inglés) sufrieron eventos psicológicamente traumáticos en el trabajo durante la pandemia por COVID-19. Para algunos, estos acontecimientos se vuelven a experimentar como recuerdos intrusivos no deseados, recurrentes y angustiosos. Se necesitan medidas simples de apoyo psicológico para reducir estos síntomas de estrés postraumático en esta población. En el laboratorio se ha desarrollado una nueva intervención para abordar los recuerdos intrusivos, conocida como Intervención de Tareas Competitivas de Imágenes (ICTI, por sus siglas en inglés). La intervención incluye una breve señal de recordatorio de la memoria, luego una tarea visoespacial (juego de Tetris® que utiliza instrucciones de rotación mental durante aproximadamente 20 minutos) que se cree que interfiere con la imagen de la memoria traumática y reduce su intrusión. La intervención se ha adaptado y evaluado recientemente en un ECA (ensayo controlado aleatorizado) con trabajadores sanitarios suecos (identificador de ClinicalTrials.gov: NCT04460014). Aquí nuestro objetivo es explorar cómo los participantes del ECA experimentaron la nueva intervención.

**Objetivo:** Explorar cómo los trabajadores sanitarios que trabajaron durante la pandemia por COVID-19 experimentaron el uso de una intervención breve para reducir sus recuerdos intrusivos del trauma relacionado con el trabajo.

**Método:** Se utilizó un análisis fenomenológico interpretativo para comprender en profundidad las experiencias vividas por los trabajadores sanitarios que utilizaron la intervención. Siete participantes del ECA fueron entrevistados por un investigador independiente sin conocimiento previo de la intervención. Las entrevistas se realizaron por teléfono y se transcribieron palabra por palabra.

**Resultados:** Se generaron cuatro temas generales, ‘Desencadenantes e imágenes problemáticas’, ‘Cinco preguntas sobre el apoyo: qué, cuándo, por qué, por/con quién, para quién’, ‘Recibirlo, creerlo y hacerlo’ y ‘La intervención. – una ayuda diferente’, los dos últimos incluían dos subtemas cada uno. Los resultados reflejan similitudes y diferencias de los participantes en sus experiencias vividas de recuerdos intrusivos, medidas de apoyo e impresiones y efectos de la intervención.

**Conclusión:** Las experiencias de los trabajadores sanitarios con el nuevo ICTI reflejan una evaluación prometedora de la intervención como una posible medida de ayuda para reducir los recuerdos intrusivos después del trauma, y nos brinda una comprensión detallada de las necesidades de los trabajadores sanitarios, con sugerencias sobre cómo adaptarse para una implementación futura.

## 1. Introduction

The psychosocial effects of coronavirus disease 2019 (COVID-19), during and after the pandemic, have been widespread around the globe and across vulnerable groups, including healthcare workers (HCWs). HCWs were on the frontline of the pandemic and experienced a significant increase in work demands due to the high number of infected patients, lack of staff, lack of knowledge about the disease, lack of treatments, and high level of exposure to the disease (McGlinchey et al., 2021). While being responsible for the treatment of infected patients, HCWs themselves were at risk of virus transmission, and witnessed an inordinate amount of human suffering (Hegarty et al., 2022). A traumatic event is defined as ‘exposure to actual or threatened death, serious injury, or sexual violence’ (American Psychiatric Association, 2013), implying that witnessing human suffering can be regarded as a potentially traumatic event. Furthermore, cumulative trauma exposure, as in repeated exposure to traumatic events, increases the risk of post-traumatic stress disorder (PTSD) (Breslau et al., 1999; Mealer et al., 2009). Several studies have indeed reported an increase in psychological symptoms, e.g. stress, anxiety, and a high prevalence of mental disorders such as depression and PTSD, among HCWs during the pandemic (Ezzat et al., 2021; Greene et al., 2021; Marvaldi et al., 2021). Both a full-scale PTSD diagnosis and subclinical symptoms of traumatic stress can have a major impact on overall functioning (Bonanno et al., 2010; Galatzer-Levy et al., 2018) and the ability to work in a healthcare setting (Iyadurai et al., 2023).

There is a need for simple interventions to reduce the symptoms of post-traumatic stress (such as intrusive memories) in HCWs, which can be fitted into their limited time between shifts and irregular working hours. Moreover, not all have the possibility to access traditional psychological treatment for trauma, e.g. therapy or counselling, and many face barriers including stigma, limited resources, and expenses (Kantor et al., 2017). Currently, many psychological programmes in general fail to consider long-term solutions for HCWs’ well-being and cannot ensure the sustainability of the intervention, e.g. whether an intervention could be applicable outside the framework of a humanitarian crisis (David et al., 2022). To improve current psychological support measures, interventions that are adaptable to any setting are imperative (Holmes et al., 2020). Furthermore, many current mental health interventions show limitations, e.g. having a poor dissemination and uptake (Cairns et al., 2021; de Beurs et al., 2017; Schleider, 2023). One driving factor for such limitations may be the lack of consequent involvement of the end users throughout the development, assessment, and implementation of interventions. That is, for many interventions that have been evaluated to ensure efficacy, their dissemination effectiveness may not have been evaluated in the same capacity. Schleider (2023) describes that there is a risk of building interventions that will not be accessed or utilized by the intended population. Input from end users, also labelled the ‘experts by experience’ (EBEs), can be seen as vital in the development of lay-delivered psychological interventions, but the exact strategy of how to implement the involvement of EBEs remains unclear (Owen et al., 2022; Schleider, 2023).

One attempt at creating a user-friendly, theory-driven simple intervention that addresses a single symptom (intrusive memories of trauma), and at the same time attempts to address some of the shortcomings of existing mental health interventions in general, is the imagery-competing task intervention (ICTI). It has been developed and piloted during the past decade in the laboratory and with various clinical populations (e.g. Holmes et al., 2009; Iyadurai et al., 2018; James et al., 2015; Kanstrup et al., 2021a; Kanstrup et al., 2021b; Kessler et al., 2018), including nurses (Singh et al., 2021). The intervention aims to prevent or reduce a single symptom of post-traumatic stress, namely intrusive memories (i.e. recurrent, involuntary, and distressing memories of a traumatic event or events) (American Psychiatric Association, 2013), a key symptom of PTSD. The intervention procedure includes a brief memory reminder cue, then a visuospatial task (Tetris^®^ gameplay using mental rotation instructions, for a duration of approximately 20 min), which is thought to interfere with the traumatic memory image and reduce its intrusiveness, i.e. to stop the trauma memories from popping up involuntarily. Prior to selecting which specific intrusive image to target first with the intervention procedure, participants create a bullet list of brief descriptions (approximately six words per intrusive image, to pinpoint content), and they can subsequently return to this list to select additional images to target. The intervention has so far been delivered via in-person sessions with a trained facilitator (e.g. Kanstrup et al., 2021a) and in a digital format with remote support from trained facilitators (e.g. Iyadurai et al., 2023). See Iyadurai et al. (2019) for details on the theoretical rationale for the intervention, and the rationale for targeting intrusive memories specifically.

In this qualitative interview study, we included our EBEs (i.e. HCWs) to help to shed light on experiences of using the ICTI. More specifically, we sought their views to understand how to adapt, use, and improve the intervention for any further development and utilization. By including end users, we aim to ensure that the intervention aligns with the needs of HCWs and is deemed applicable for future use after a person has experienced any type of work-related traumatic event. The objective of this study is thus to explore how HCWs who worked during the COVID-19 pandemic experienced the use of a brief intervention to reduce their intrusive memories of work-related trauma.

## 2. Method

### 2.1. Study population and recruitment

Participants in the present interview study were HCWs recruited from a two-arm parallel randomized controlled trial (RCT): ‘Simple cognitive task intervention after trauma during COVID-19 in hospital staff “EKUT-P” – a randomized controlled trial’ (ClinicalTrials.gov identifier: NCT04460014) (see study protocol paper: Singh et al., 2022). The primary aim of the RCT was to determine whether a simple cognitive task (i.e. ICTI), compared to an attention placebo, after a work-related traumatic event, can reduce the number of intrusive memories reported by HCWs at week 5 (using an intrusive memory diary). Participants (total *n* = 164) could be allocated to a simple cognitive task intervention (ICTI) or an attention placebo arm (listening to a podcast). For eligibility criteria for the RCT, the reader is referred to the study protocol (Singh et al., 2022). Each arm included a researcher-guided session where the participant would complete their assigned task together with a researcher via a telephone call. The RCT was conducted fully remotely and data collection was conducted via the electronic platform SMART-TRIAL between September 2020 and October 2022.

A subsample of participants from the RCT was contacted to participate in the interview study. Participants in the RCT who had already completed their final 6 month follow-up were not invited to take part in the current interview study. In line with the interpretative phenomenological analysis (IPA) guidelines, we aimed to include a relatively small sample size (Smith et al., 2021), e.g. a maximum target sample size of 15 people. Interviews were conducted remotely via telephone. Eligibility criteria for participation in the interviews, extending from the wider RCT, were as follows: allocated to the intervention arm, completed the week 5 diary, and not yet reached the study end (i.e. non-completion of the 6 month follow-up questionnaire). Invitations were sent twice via e-mail (through the RCT study e-mail address) to participants who fulfilled the above criteria. Thirty-three participants from the full intervention sample (*n* = 73) were contacted by the research team between 15 June 2022 and 19 August 2022. Nine participants replied and showed interest in participating in the interviews. When proceeding to schedule the interview, one participant did not reply and one participant declined to participate owing to a personal life event, leading to a final number of seven participants being interviewed.

The time between the researcher-guided session, i.e. the ICTI, and the interviews ranged between 41 and 147 days (*M *= 104.71, *SD* = 34.58 days) among the participants. Informed consent had been collected before their participation in the RCT. The informed consent sheet included an optional yes/no item (‘I agree to being contacted again by the research group to be asked about eventual participation in other studies in the future’). All participants who were contacted for the interview study had already replied ‘yes’ to this item, and later confirmed their participation in interviews in response to the e-mail invitation.

### 2.2. Participants

Seven participants (five female, two male) aged between 28 and 51 years (*M* = 40.29, *SD* = 8.58 years) were interviewed. Yearly income varied between 0–249,999 SEK and ≥ 550,000 SEK [compared with the average yearly income for HCWs in Sweden, 471,132 SEK (Vårdförbundet, personal communication, 2023)]. Five participants were nurses and two were assistant nurses. They originally came from varying types of workplace [e.g. intensive care unit (ICU), elderly care, and paediatrics]. At the time of the baseline questionnaires assessed in the RCT, they had worked in healthcare for 3–26 years (*M* = 12.14, *SD* = 8.86 years). Three participants were still at the same workplace as they had been prior to the pandemic, while the others had changed workplace due to either dissatisfaction at the current workplace or progress in their career.

### 2.3. Interpretative phenomenological analysis

We used the qualitative analytical approach of IPA, which is commonly used in health and social psychology but also other bordering disciplines. The aim of IPA is to capture participants’ ‘lived experiences’ by examining detailed reflections of a specific phenomenon that the participants have experienced (Smith et al., 2021; Smith & Osborn, 2015). This aligns with the focus of the study, i.e. gaining a detailed understanding of how to adapt, use, and improve the intervention through the participants’ accounts and their lived experiences. IPA emphasizes open-mindedness, as the outcomes are governed by the participant’s own words and not by theoretical preconceptions. A small sample size and semi-structured interviews are common key components in IPA.

The interviews were conducted by LJ, an ICU specialist nurse and independent researcher (i.e. not part of the research group responsible for the wider RCT). The interview template (see Supplemental Material) for the semi-structured interviews was created by MK with input from LJ before the start of data collection. Three topics were covered in the interview template: (1) participants’ experiences of the ICTI; (2) participants’ work experiences during the pandemic and prior to participation in the RCT; and (3) participants’ experiences of other support measures at work during the pandemic. The questions were adapted from a parallel interview study conducted with ICU staff in the UK (*n* = 16) (Patel et al., 2024), who had participated in a randomized trial where they received the same intervention (ICTI) but using another digital platform (Iyadurai et al., 2023). The interview template was piloted by LJ in a roleplay together with an experienced facilitator of the intervention (MK), prior to the first interview. Questions were open-ended and, when needed, the participants were given prompts, such as ‘how so?’ or ‘tell me more’. The content/direction of the interview was flexible depending on the accounts of each specific participant, while still adhering to predetermined topics.

LJ had no prior knowledge of the intervention, the participants, or their RCT data. Similarly to the participants, LJ, a critical care nurse, had worked in healthcare during the COVID-19 pandemic (first line manager in an ICU between 2018 and 2021). The participants were informed of this in the initial e-mail invitation and during the introduction of the interview. Furthermore, LJ had prior experience of conducting qualitative and phenomenological research in a healthcare setting. EAH, MK, and SAP had all worked as researchers on the RCT, and MK and SAP had facilitated the intervention to participants. MK and SAP performed investigations including the intervention component with two participants each from the current study sample, i.e. a total of four out of seven participants. EAH and MK are both clinical psychologists with experience of training facilitators, including, but not limited to, the RCT. MK had previous experience of IPA research.

### 2.4. Procedure

Interviews were scheduled via telephone or e-mail correspondence with LJ, set at a convenient time for the participant, and with one interview per participant. The interviews were conducted between June 2022 and September 2022. SAP reflected together with LJ between the interviews to allow possible improvements throughout the process. Examples of such feedback included checking the quality of the audio recording each time, and slowing down questions/prompts so as not to ask more than one question at a time. Prior to recording, participants were informed by LJ that the purpose of the interview was to provide an opportunity to describe, in their own words, how they experienced the intervention and any support provided. The interviews were conducted in Swedish and carried out remotely via telephone. They were recorded using an external device, the Olympus digital voice recorder VN-541PC. Audio files were then saved on a secure server. The duration of the interviews ranged between 22 and 58 min (*M *= 37.37, *SD* = 12.49 min), depending on participants’ accounts or until data saturation (i.e. the themes were answered adequately according to the interviewer). Demographic data (see Section 2.2) was collected from the RCT (e.g. gender, age, yearly income) and from the interviews (e.g. workplace).

### 2.5. Data analysis

The interviews were transcribed verbatim by SAP via oTranscribe (https://otranscribe.com/), followed by a credibility check by a research assistant. Confidentiality was ensured by removing potentially identifiable information, e.g. names and geographical locations, from the transcripts. Participants were given a pseudonym. To ensure accurate interpretation of the participants’ words, Swedish was primarily used in the analysis, until the labelling of group experiential themes (GETs) and onwards. Following the process outlined in Smith et al. (2021), the analysis was performed case by case, with one participant (i.e. one interview) at a time. The process was initiated with exploratory notes and rereading of the transcript. Then, as per the most recent revisions of IPA (Smith et al., 2021), experiential statements were constructed; this was followed by clusters and labelling of subthemes and superordinate themes, thereby constructing personal experiential themes (PETs). After PETs had been constructed for each person, GETs were formulated through an iterative process by comparing each PET.

Throughout the whole process, the transcripts were revisited to make sure that the participants’ own words were appropriately interpreted and taken into consideration. Furthermore, as per the recommendations of IPA (Smith et al., 2021), physical copies of the material were printed to allow closer inspection of data, e.g. when writing exploratory notes and initial experiential statements (which were later digitized and revised), and an open-mindedness to different possibilities of linkages, e.g. as a first step towards creating clusters for PETs and GETs. The analysis was conducted with NVivo 14 to ensure traceability. Reflective discussion took place between SAP and MK to tackle emerging questions throughout the process. The supervision meetings were conducted approximately weekly, in-person or remotely via telephone or Zoom for the duration of the analysis period (August–November 2023). SAP kept reflective notes throughout, starting at the introduction to IPA to conducting the analysis, to document thoughts and reflections surrounding the method and the content of the interviews.

## 3. Results

During the interviews, participants described their extreme and unprecedented situation at work during the pandemic. This served as an important contextual backdrop for the present analysis, and their experiences were in line with other recent literature, e.g. reports of ethical stress (Hegarty et al., 2022; McGlinchey et al., 2021). Participants described work as chaotic and stressful, and expressed their disappointment in management for not meeting their needs. However, such accounts of wider workplace experiences during the pandemic were beyond of the scope of this study and therefore not included in the present analysis.

Data analysis revealed four GETs, two of which included two subthemes each. The first theme, ‘Triggers and troublesome images’ is constituted by participants’ terrible experiences, of which they had not been able to let go. This was followed by the theme ‘Five Ws regarding support – what, when, why, by/with who, for whom?’, which covers participants’ experiences of the support offered, or not offered, to them in relation to the strains of the pandemic. The third theme, ‘Receiving it, believing it, and doing it’ is constituted by two subthemes: ‘Reaching the right people in the right way’, where participants described how different channels can be used to reach the target population differ in terms of their credibility, and ‘What’s in it for me?’, where participants explored the gap between believing in the intervention and actually doing it. Lastly, the theme ‘The intervention – a different kind of help’ is constituted by two subthemes, ‘The parts and the whole’ and ‘A different tool’, and delves into the participants’ lived experiences of using the actual intervention. The names used in the following sections are pseudonyms.

### 3.1. ‘Damn it, I am back there’ *–* triggers and troublesome images

Participants described being troubled by images that would not go away, and which to some extent still remained at the time of the interviews. Trivial things in the everyday work context triggered these images. Anna talked about how putting on a protective equipment cap made her travel back in time to the pandemic and her feelings from when they put on caps just before entering the ward: ‘so it was like that occasion like “damn it I am back there”’. Their flashbacks could also be of patients, as described by Jenny, ‘so when we were taking his blood samples he started bawling, like tears were streaming’, and Magnus, who would awake at night to images of ‘saw like the mother scream and stand there with the Ambu bag and pumping and it, ah it was really really horrible’. These intrusive image descriptions depict vivid situations in which they as HCWs were unable to provide any relief to these suffering patients and relatives. A common denominator for people in the HCW profession is to help people, which stands in stark contrast to the content of the images with which these participants were burdened.

Such images and the related feelings sometimes remained in an endless loop in participants’ minds, or were really stuck, as for Maria, who could see herself from the outside, walking a certain route through the wards.

It is stuck in my mind when I am walking all this way, it is very surreal. There you feel ah, feel like, like seeing from the outside like, and it was also one like ‘ooooomyyyygod’. That feeling and image is stuck.

Furthermore, the quote below shows the extent of the distraction that their intrusive images could cause. Anna exemplifies this when comparing different triggers in an attempt to make sense of the impact on everyday tasks:

The brain has to process too, but it still feels like well – yes I can accept that when I put on protective equipment and stuff like that. But I have mixed medicine for 12 years and I will continue to do so for 20 years more (…).

Having intrusive memories due to the protective equipment was, according to Anna, more justifiable (‘yes I can accept that’), which could be attributed to the equipment itself, for them, being more exclusively associated with the COVID care. However, mixing medicine was an established everyday routine prior to the pandemic that will continuously be repeated; therefore, experiencing this as triggering was perceived as more problematic. Combining the perspectives of Anna and Maria portrays an inner conflict, when the wish to move on and not to dwell on the events or images was hindered by being ‘back there’ and the image being ‘stuck’.

The participants described lacking tools for dealing with their symptoms effectively, i.e. lacking strategies to process or move on. Camilla expressed, with emphasis, a sense of not knowing what to do, or even fully realizing what she was grappling with: ‘Wow, did I experience this situation? How do I cope with that? Like how will I be able to process these memories?’, and Jenny noted that talking to others about the subject felt ‘not shameful, but eh, like a bit troubling for others’. Similarly, Daniel said that he has ‘not really talked to anyone about it now either’ (i.e. after performing the intervention; this differs from other participants, who also did not talk about their symptoms initially, but were able to do so after they had participated in the study), indicating that these experiences are a burden that they carry alone. Altogether, the participants’ experiences illustrate the loss of control when the images take over, rendering one powerless to the visual imagery and feelings at the time of the event. The powerlessness extends to taking the first step to moving on, and how, and where do you even start?, i.e. the feeling of ‘I want to move on but I can’t’. This first step and other experiences with support are explored further in the next theme.

### 3.2. Five Ws regarding support – what, when, why, by/with who, for whom?

Here, participants differed in their experiences and the needs they had had, from stating that employers offered no support at all (*n* = 3) to describing that there was plenty of support available, and some using such support, others not. Magnus said, ‘we never got anything supportive in COVID wards, and we hardly got support in [original workplace] either. Like how you felt, and nothing, absolutely nothing, at COVID [ward]’. Here, the frustration at the lack of support is strongly tangible when the bare minimum of asking about their well-being was not extended by the employer, whereas Daniel and Hanna both felt that they had been offered good resources for support but that they themselves were not up to it during that specific time: ‘ … they can’t do more than offer it. Then if I don’t accept it, that is up to me’ (Daniel); ‘ … resources were available, it just that when, during first wave when I really maybe needed it, I was not receptive to it’ (Hanna). These two participants describe being more or less in the right place at the wrong time to explain why they did not utilize the support offered; while the need may have been the most pressing at that time (‘when I really maybe needed it’), for them it was not a plausible option. Moreover, had one used the support, it is not certain that it would have made a difference since the commitment was not there (‘I was not receptive to it’). In addition, Daniel suggested that some of the responsibility in getting support lies within oneself (‘that is up to me’). Regardless of whether what is offered is the right fit, it could be beneficial to try it in comparison the alternative – nothing. No blame seems to be put on the employer by Daniel (‘they can’t do more’), indicating an understanding of the perspective of the employer and their potential constraints.

In the lack of appropriate or suitable support measures, some participants turned to their colleagues, which was sometimes already an established source of support prior to the pandemic. Anna described how the staff themselves created routines (e.g. debriefings) to alleviate the burden of their experiences and confide in each other.

But partly we had – we did so that we – after every shift we sat in the staff room a quarter, twenty, and like just talked a little so that one … If it was like something very difficult, we did have some quite difficult integrations or [unintelligible] cardiac arrests, you raised it to avoid bringing it home with you and talking to colleagues.

When a sudden or difficult event occurred, it needed to be processed, according to Anna’s description; holding on to a difficult event meant carrying the burden for longer than necessary, and ‘bringing it home’ could in itself be a liability and cause detrimental effects.

On the other hand, when provided with ‘group interventions’ or in discussions among colleagues, Hanna noted that the long-term consequences of work-related traumatic events and the individual experiences following this were seldom raised.

So it very much strengthens the group to do stuff like counselling and reflection together and it is absolutely necessary. But when you have like your own issues, like me or these intrusive memories, then they are not brought to light.

The sharing of work experiences could serve as a support for one other and strengthen the unity among the group, but by itself it was not enough. Hanna’s own personal needs were still neglected and consequently had a negative impact on her well-being. Furthermore, Camilla said that even when there was a standardized procedure with debriefings, the group fell through during the pandemic, ‘But there was no space, people were so … what can I say, people are so tired’. On top of caring for patients, they also had to care for and support each other, all of which eventually accumulated until it was too much. The different experiences of support that the participants describe above demonstrate the different needs of each participant, indicating that a ‘one-size-fits-all’ solution may not be feasible.

### 3.3. ‘Receiving it, believing it, and doing it’ – ‘Reaching the right people in the right way’ and ‘What’s in it for me?’

For the first subtheme, ‘Reaching the right people in the right way’, in terms of credible channels who can communicate about a support intervention, management/employers were stated as reliable sources by Hanna: ‘it would have had to go via the employer’. In contrast, social media was described as less credible by Daniel: ‘If I had just read about it in social media then I would have believed that it was someone … just something that someone had been sitting at home and like made up’, but if the sender is credible (e.g. university hospital official social media channels) it is more likely to be perceived as relevant. Magnus:

There is a lot of crap going on in social media for example, but when you are met with something like it is, has, well let’s say if [hospital name 1] posts or [hospital name 2] post or something else like that.

The repeated notion of social media as a dubious source of information illustrates the sensibly cautious approach of the particular, well-informed population of HCWs. The professional knowledge of an HCW, along with personal experiences of ‘what is out there’ and disseminated online, including disinformation or false promises of remedies, shape which types of channels are perceived as reliable.

When participants mentioned the intervention to colleagues, some participants were met with interest in using the intervention, indicating that a peer-to-peer approach may be a beneficial strategy.

( … ) because we were a few colleagues that all around that felt ‘But God, this is something I should do’. (Hanna)

And I have done – I have talked with a few colleagues and such and they said that ‘Dammit that we missed it’ and like so. Because I sent them a link to someone but they did not care about it and they did not have time and stuff but it actually so they regret it now. (Magnus)

Evidently, the occurrence of intrusive memories affected not only Hanna and Magnus, but also their colleagues. However, a proper recognition of the symptom and knowledge that it could potentially be addressed seem to have been lacking, as their colleagues’ expressed surprise after learning about the intervention (‘But God … ’) or regret at having missed it. The colleagues’ reactions also reflect on the earlier struggles mentioned in ‘Five Ws regarding support’, e.g. not being receptive to utilizing support when offered (‘they did not care about it’). Expanding from the current aim, Magnus suggested that ‘the right people’ could include non-healthcare staff, ‘And maybe you don’t need to be healthcare staff either to have experienced something terrible’. Presumably, as a result of either personal or work-related experiences, or both, Magnus understands that a traumatic event and the occurrence of intrusive memories thereafter could be applicable to others as well and that there is a general need for help measures.

In ‘What’s in it for me?’, participants described their initial impressions of the intervention, which included feeling sceptical as to the potential effects, i.e. the reduction of intrusive memories. This attitude was later dropped post-intervention, when they noticed a change in their symptoms. For example, Anna said,

Like that it felt ridiculous but it seems to work and then it is worth it. Because it really felt like, when she said it at first you just ‘So this is so incredibly ridiculous, what am I supposed to do with Tetris?’

Similarly, Daniel stated ‘Like when I first heard about it it sounded a bit silly but I thought it worked pretty well’. Although Anna noticed an effect, there still seems to be some doubt as to the intervention’s soundness (‘it seems to work’). Looking at the wording the participants use, i.e. ‘silly’ and ‘ridiculous’, hints at something childish or inappropriate instead. The notion of a legitimate intervention including Tetris raises a lot of doubt, probably because it diverges from typical support measures, e.g. psychological therapy.

The participants noted several aspects to consider to motivate others to utilize the intervention and to manage their expectations. First, it is important to understand the basis of the intervention and the expected results, as described by Magnus, ‘It is important to know what it involves, what the payoff – hopefully – will be for the one that participates, goes through it. So that one hopefully presumes that they are motivated’, and Daniel, who said, ‘But I needed someone to explain the basis of it’, indicating that they themselves could not gain this understanding when reading about the intervention. This presents an example of the type of concerns that participants may have at the point of introduction to the intervention – the need to be assured that an effect can be expected and an explanation as to why, and thus a potential challenge to address.

Secondly, participants noted that the person or organization providing the intervention has to show that they are trustworthy. Similar to what is mentioned above, with credible sources informing them about the intervention, the actual facilitator has to ascertain that they are perceived as reliable. Magnus describes how he values whether there is a genuine intention to help from the person delivering care, ‘Then I felt like more of a relief to actually have someone that took this seriously. That’s how I function at least so’. For Magnus, the interaction with the researcher was referred to as ‘a relief’, perhaps as opposed to earlier experiences with support measures or instances where he may have been let down. This highlights the importance of trust, in trusting that ‘you will do well by me’, especially when asking for help, which may put a person in a vulnerable position. Following on this trail, Maria talked about the importance of knowing who the facilitator is and how one’s personal information is processed. ‘So it was important to me, because it, that you – ah that you know who you are talking to and know where the things end up and that it is a safe company like or whatever you say’. To be guaranteed that personal information is responsibly taken care of again reflects that this is a cautious and well-informed population, who are aware as consumers of what to look out for.

### 3.4. The intervention – a different kind of help

The first subtheme, ‘The parts and the whole’, included participants’ experiences of the intervention on different levels – they addressed both specific aspects of the interventions (e.g. making the list, the gameplay, the monitoring of the symptom) and the practical implications of a self-guided intervention instead of a researcher-guided intervention. Anna described the list of intrusive memories as a challenge in itself because of the distress it caused, ‘It is not that much fun in my world, “ah let me look at and think about something that I find distressing”’. This was echoed by Hanna, although she also recognized the importance of the process: ‘I understand that this is what you need to do, that it is kind of a processing in itself (…) Then it was like difficult and distressing there but that weighs against that I understood that it was crucial’. Naturally, many people may wish to avoid distressing experiences such as briefly bringing their trauma to mind, but, as emphasized by Hanna, the benefits need to outweigh the cons. Once more, there needs to be an assurance that there will be benefits, or in other words a ‘payoff’, as stated by Magnus in ‘What’s in it for me?’ Across participants, the gameplay was received very well, with them referring to it as fun, easy, and nostalgic; for example, Daniel stated, ‘I like videogames so Tetris is fun’; whereas the number of data collections per day on the number of intrusive memories experienced in each time slot was deemed as excessive, as Maria said: ‘I do surely think that it may be a little too – like three times per day, if we say it like that. Maybe it would have been enough with once per day’.

The perceived usefulness of the intervention as a self-guided tool varied among the participants. Magnus described that he had experienced additional symptoms/incidents a little while after the initial use of the intervention, ‘and then I like followed these instructions and played Tetris so it worked like kick-ass’. In contrast, Camilla stated that ‘I get like – these pretty intrusive memories occur when I meet a patient in a difficult situation or a patient that looks the same or patient – but I can’t pick up a Tetris thing then’. If the intervention were to be of any use, Camilla thought it needed to be used as soon as the intrusive memories occurred, which was not possible for her (‘but I can’t pick up a Tetris thing then’), whereas Maria verified it as easily adaptable to everyday life and that it did not have to be used at the exact moment when the intrusive memory occurred:

Yes [agreeing to the possibility of independent usage], and you played at your own pace so that things happen in life all around so that you, maybe you can do that [the intervention] more in, a bit further ahead maybe.

The difference between Camilla and Maria was whether they found the intervention to be limiting or not; the former believed that the intervention could only be potentially effective in specific settings/situations, and the latter saw the freedom associated with the intervention and how its use could be flexibly fitted into her life situation. Notably, Camilla was the only participant in this sample who had experienced no reduction in her intrusive memories.

As for utilization without a researcher, when imagining performing the intervention alone with the current instructions and setting, Magnus said, ‘Yes, I’m sure of that. Absolutely’. In contrast, Daniel and Hanna expressed a need for a facilitator when carrying out the intervention as it otherwise would result in incorrect execution of the different steps or a loss of motivation to complete the intervention fully. ‘I actually don’t know what I would have needed to do it in the correct way. But … no, I probably would have needed someone to give me instructions’ (Daniel); ‘I believe mostly that you, that you don’t follow-up or that you don’t – that you just do it with one image like for an example’ (Hanna). Similarly, Anna noted one specific problem regarding self-usage, namely that it would be challenging to ‘get hold of that memory’ that was to be specifically targeted, but that the other components of the intervention would be easy. These experiences tap into the experience of distress associated with briefly listing the images to be targeted and briefly bringing one such image to mind (memory reminder cue). In an attempt to avoid distress, one may refrain from repeating the intervention for the remaining images and consequently prevent the reduction in the remaining intrusive memories.

The subtheme ‘A different tool’ covers overarching aspects such as the applicability of the intervention for different people, the value of this kind of approach, and the effects that the participants did or did not experience. In terms of effects, several participants noticed a reduction in the intrusive memories. Daniel said, ‘So I don’t have, they don’t show up as often any more. So they definitely have and they are not as intrusive should I say, not as distressing any more’, and some participants described how the distress levels related to the traumatic image had been reduced, e.g. Jenny: ‘ … so I can still see like the images in front of me but it is not as, it is not as emotionally charged kind of. It is not that terror’. Daniel’s and Jenny’s quotes highlight the change from the dissociation and powerlessness when the images popped up to still being able to stay present as you face the image. One exception to this was Camilla, for whom the intervention did not have any effect at all. She contributed it to her personality traits. ‘ … I’m a little that kind of person, I am pretty calm in these acute situations, I am pretty strategic. So I think that Tetris maybe, at that level it is pretty difficult but I don’t think so’. The presumption was that there was a threshold to whether or not the intervention had an effect, i.e. that the game had to be challenging enough to interfere with the trauma image (‘that level it is pretty difficult’), and, in her case, this threshold was not high enough for her.

The intervention was described as being a different type of option, more specifically a missing piece to the types of measures available. Daniel stated, ‘I thought it was really good, I mean, like, for us who don’t get on so well with counselling it’s a really good thing. That there’s an alternative’. The requirements of therapeutic dialogues, to ‘open up’ to a stranger, are implied to be a challenge for Daniel and his personal needs. In comparison, the intervention, clearly deviating from these requirements, is perceived as a relief. In comparison to other types of help measures, Jenny said that she preferred the intervention over therapy. ‘No it feels more like, for me it feels like it actually targeted like the emotions more. Because I have been to therapy as well, that felt more like you were ruminating rather than moving on like’. Although the format of therapeutic dialogues could provide a match, there was no assurance that it would have an effect, as acknowledged by Jenny. The traditional help measures, specifically therapeutic dialogues, were neither compatible with nor sufficient to meet the needs of Daniel and Jenny. Several of the participants thought that the intervention would be useful for those who do not want to talk to someone, reflecting on the examples above, although almost unanimously the participants also emphasized that the intervention may not necessarily be the right fit for everyone. Such individual differences are not necessarily problematic – as Camilla described it, ‘So I think that we, we are really different as humans, maybe we should have different tools’.

## 4. Discussion

The aim of the current interview study was to explore how HCWs who worked during the COVID-19 pandemic experienced the use of a brief intervention to reduce their intrusive memories of work-related trauma. Themes generated from the seven interviews covered participants’ experiences of intrusive memories, support, initial impressions of the intervention, and the lived experiences of using the intervention.

As described in the theme ‘Triggers and troublesome images’, the occurrence of intrusive memories was distressing in itself, but it also had a palpable effect on the functioning of the participants. They were disrupted in their work tasks and, for some, the effect lingered beyond as well (e.g. images appearing in nightmares). Similarly, for HCWs in the UK, participants had experienced changes in cognitive and emotional coping due their intrusive memories, changes that were later reduced post-intervention (Patel et al., 2024). Previous studies show that a high frequency of intrusive memories is associated with a stronger impact on functioning (Holmes et al., 2017; Singh et al., 2021), which may be reflected in the current study sample as well as among the UK staff (Patel et al., 2024).

The lack of dialogue about intrusive memories, and the delayed realization of needing help upon acknowledgement of intrusive memories, illustrates the stigma associated with experiencing sequelae from traumatic events and consequently the lack of knowledge about the consequences of these experiences. Exemplified in ‘Five Ws regarding support’ is the notion that a debriefing at work should be enough as a processing tool after a traumatic event. Although the exact focus and content of the debriefings that our participants described are not known, e.g. whether it was an operational debriefing, a psychologically focused debriefing, or a critical incident stress debriefing, it is worth noting that the last two of these are not recommended according to international guidelines (National Institute for Health and Care Excellence, 2018; PTSD: National Center for PTSD, 2022; Swedish Agency for Health Technology Assessment and Assessment of Social Services, 2019). In terms of support, any admission of more serious actions being required was not acknowledged until it became evident for Anna, similarly to other participants, that it was not enough. Repeated instances of traumatic events may, together with the increased workload, the lack of support, and the inability to provide good care (i.e. ethical stress; see Svantesson et al., 2022), have exacerbated the stress and duress under which HCWs were placed. Moreover, a conflict presents itself when an HCW, who has a profession known to help people, is unable to fulfil this exact purpose.

Our participants’ stories tell us the how varied individual needs can be. Anna was comfortable confiding in her colleagues, Hanna was present in group settings but did not share her personal struggles, and Daniel never expressed his symptoms to anyone in his private or work life. Thus, a broad array of complementary approaches may be necessary to ensure that individual needs are met. This was summarized neatly by Camilla: ‘So I think that we, we are really different as humans, maybe we should have different tools’. In terms of interventions, the ICTI fulfils certain features that others may lack. It provides a brief, self-administered intervention that is available remotely, and with the use of Tetris, is free to use at the recipient’s convenience. Furthermore, it can be repeated in the case of ongoing or repeated trauma. Participants’ narratives throughout speak to the end goal of each and every person receiving the support that they need, and having a choice in the type of help that they prefer. We credit the artist Louise Bourgeois and her works ‘Who, where, when, why, what’ and ‘The five magic words’ (see e.g. Bourgeois, 1999, 2008) for inspiration on the name for the theme ‘Five Ws regarding support’ – which are indeed magic words for carefully tailoring initiatives to provide psychological support and to consider how to make best use of support measures and maximize their impact for a given individual at a given time. Should further evidence continue to support the effectiveness of the intervention, ICTI could become part of the range of options to choose from after trauma, specifically to reduce intrusive memories. It could be used either as a complementary approach together with other evidence-based psychological therapies aimed at treating the full PTSD, or, in case no other measures are available, as a stand-alone tool to reduce the single symptom of intrusive memories (Bisson et al., 2013; Swedish Agency for Health Technology Assessment and Assessment of Social Services, 2019).

One obvious challenge, as reflected in the theme ‘The intervention – a different kind of help’, concerns the potential for scalability, i.e. the possibility of independent use. With feedback from participants, there are some easier adaptions and some more difficult ones to address. The easier adaptions include reducing the number of data collections per day. Although there are many potential benefits for participants as they monitor their own symptoms (e.g. the ability to track progress: to understand links between symptoms and specific situations), such monitoring needs to be tailored to their needs so that it does not interfere in daily life. Revising the current instructions to be more informative and presenting the rationale earlier are also simple adaptions. Regarding the components of the intervention, the gameplay itself was described as a mostly positive experience. The main concern was the memory reminder cue, along with the list of intrusive memories. Without the researcher, some participants doubted their abilities to complete and later repeat the intervention because of the distress caused by the list of intrusive memories or the memory reminder cue. Therefore, this would defeat the aim of the ICTI, to provide a scalable and remote tool, if there is a need for the involvement of a (trained) facilitator. In future iterations of instructions, key messages could be enhanced, e.g. that the hypothesized benefits (i.e. long-term reduction of intrusive memories) outweigh the cons (i.e. short-term distress). To date, no studies have been conducted with self-administration of the ICTI, which is an important next step. Similarly, the findings of Patel et al., 2024 also include some concerns and suggestions related to the independent use of the intervention. The importance of continuing to include lived experiences cannot be overstated, as it is not just about implementation aspects but also about ensuring a diversity of perspectives when building support measures (Schleider, 2023; Singh & Gatera, 2021).

Aside from their role in humanitarian crises, HCWs in critical care face highly stressful work environments (Ross, 2020; Salmon & Morehead, 2019). The International Council of Nurses warns of a global health emergency as the worldwide nurse shortage is estimated to reach 4.5 million in 2030 (Buchan & Catton, 2023). Measures are needed to provide sustainable work environments or at least to provide tools, e.g. an ICTI, to prevent or reduce threats to HCWs’ mental health and well-being when traumatic exposure is a part of their work. As raised in the subtheme ‘Reaching the right people in the right way’, the intervention could be applicable to other occupations that are subject to cumulative trauma exposure, such as firefighters and other public safety personnel (Berger et al., 2012; Carleton et al., 2019; Jahnke et al., 2016). Future studies could explore whether a broadened implementation of an ICTI is feasible.

Limitations to this study include that the homogeneity of the participants in terms of ethnicity is not representative of HCWs in Sweden overall, with one in five HCWs employed in Sweden being born outside the country (Official Statistics of Sweden, 2015). Building on this initial work, future studies should consider approaches to include a more diverse, and perhaps larger, sample to ensure a more cohesive picture of HCWs’ lived experiences. The method of data collection could also carry limitations since interviews were conducted via telephone, leaving out visual cues by participants that could have been interpreted, and thereby potentially changing the course of the interview. Future studies could still be conducted remotely to keep the threshold for participation low, but via video call instead of telephone to allow non-verbal visual cues to be seen during the interview. Translation of quotations from Swedish to English for the purpose of this article may have lost some of the meaning behind the participants’ words, an essential element in the IPA guidelines (Smith et al., 2021), although this was adjusted with a credibility check of the translated quotes to attempt to diminish the risk.

Strengths of this study include the alignment between the IPA method and the study aim to capture a richer and more detailed story of the participants’ lived experiences. Here, we were able to gain a better understanding of a few participants’ individual needs while observing general themes. At some points, participants’ stories converged with others, e.g. shared experiences of unsatisfactory support, and at others they diverged, e.g. the usefulness of the intervention. With this, we gain a nuanced picture of participants’ experiences (Nizza et al., 2021). The interviewer (LJ), having experience in the nursing profession but also being naïve to the intervention, facilitated a potential alliance between herself and the participants, e.g. both sharing experiences from work during the pandemic, but being new to the intervention approach. Throughout the analysis, continued reflective notes, together with an interplay between digital and physical material, allowed the possibility of a deeper review of the participants’ words, especially as it required more extensive scrutiny with the additional steps in between. Furthermore, with the regular supervision (MK) and logging of the research diary, the reflexivity of the researcher conducting the analysis (SAP) could be enhanced. In terms of generalizability, the experiences captured in this interview study resonated with the parallel UK study (Patel et al., 2024), thereby expanding the applicability of the experiences captured from Swedish HCWs to HCWs in the UK.

In summary, HCWs’ experiences of this ICTI approach in a Swedish healthcare setting reflect a promising appraisal of the intervention as a potential measure to reduce intrusive memories of trauma. The participants have provided us with a detailed and nuanced picture of the needs of HCWs and suggestions for how to adapt and refine our approach for future implementation.

## Supplementary Material

Supplemental Material

## Data Availability

To protect participants’ anonymity, individual participant data such as demographics are kept at the aggregate level only. Sharing of raw data and interview transcripts were not part of the ethical approval and thus these will not be shared. The interview template can be found in the Supplemental Material.
