# Supplementary material for: Exploring healthcare workers’ experiences of a simple intervention to reduce their intrusive memories of psychological trauma: an interpretative phenomenological analysis
Source: Eur J Psychotraumatol. 2024 Mar 27;15(1):2328956. doi: 10.1080/20008066.2024.2328956 (PMC10977018; doi:10.1080/20008066.2024.2328956)
Supplement: Supplemental Material [file ZEPT_A_2328956_SM3708.docx]

**Supplemental Material**

Exploring healthcare workers experiences of a simple intervention to reduce their intrusive memories of psychological trauma – an interpretative phenomenological analysis’

Sara Ahmed Pihlgren; Lotta Johansson; Emily A. Holmes; Marie Kanstrup

## Interview template

**INTRO:** **Briefly about who you are, and where you currently work - role, type of organisation - and where you worked during the pandemic (type of organisation).**

**AREA 1: The specific intervention you received in the EKUT-P study** - in the EKUT-P study, you were instructed on an intervention involving the game Tetris.

- **THE INTERVENTION**

**What did you think of the intervention? Your experiences?**

**How would you describe the different steps of the intervention, in your own words - i.e. what did you do? How did you do it? How would you describe it to a colleague, if you were to help someone else use the intervention? Please describe in a way that we could use straight away!** (*Prompts and follow-up questions/deepening if needed e.g.,*

- How was it to use it when you first learnt how to do it, with the support of the researcher on the phone? Easy/helpful? Difficult/stressful? How can it be improved? How did it work to use the intervention in your daily life? Are there any aspects of the intervention that facilitate future use if needed/make you refrain from future use?
- How did it feel to use a game as part of the intervention?
- Did you ever do the intervention on your own/independently? What was it like?
- What do you think it would be like to use the intervention only as self-help/independently, with only written/video instructions? What would be needed? What would be helpful?
- How would it feel to make the 'list' of your intrusive memories on your own? What would you need to do this properly?
- How do you think this intervention compares to other support interventions - e.g. psychological support, medication? Advantages/disadvantages? Independence vs seeing a counsellor?)
- **THE SYMPTOM**

**How did it feel for you to talk about intrusive memories before you joined the study - and how does it feel now? How could we make it easier for health professionals to talk about this symptom?** (*Prompts and follow-up questions/deepening if needed e.g.,*

- How would you describe 'intrusive memories' in your own words?)
- **FUNCTIONAL ABILITY**

**Have you found that the number of intrusive memories changed for you after doing the intervention? If so, has this affected your ability to function in everyday life, at work? What is the difference from before? Any differences in functioning in other respects - e.g. social relationships?** (*Prompts and follow-up questions/deepening if needed e.g.,*

- Any other changes that you associate with the intervention? In your mood, behaviour, work? Relationship to the specific memory itself - and behaviour/functioning?)
- **IMPLEMENTATION - future dissemination/use**

**Would you recommend the intervention to a colleague or friend - what would you highlight? If not, for what reason?** (*Prompts and follow-up questions/deepening if needed e.g.,*

- How would you feel about showing a colleague or friend how to use the intervention?
- How do you think information about the intervention could be disseminated - through which channels/in which ways? What do you think colleagues would prefer?
- Do you think there are any challenges/barriers to disseminating this intervention/implementing the intervention in health care organisations? How could we address these?
- Do you have any other ideas on how we could go about disseminating info? What channels/people/places/?!
- Any other comments on the study/intervention?)

**AREA 2: Before doing the intervention, tell us about the difficulties/stress you experienced at work during the pandemic - and how this affected you*?*** *Situations that led to intrusive memories, stress and discomfort, relationship between e.g. organisational factors/resources and individual symptoms (intrusive memories, post-traumatic stress, ethical stress*). (*Prompts and follow-up questions/deepening if needed e.g.,*

- Ethical stress - i.e. insufficient resources to provide good care - was this something you experienced during the pandemic? If yes/no - tell us more!)

**AREA 3: What various other support or assistance measures have you been offered/participated in/or chosen to refrain** **from during/after the pandemic?** (e.g. *psychological support, group reflections, ethics conferences, etc*.) (*Prompts and follow-up questions/deepening if needed e.g.,*

- Have these been offered to you through your employer? Or from another source? What are your thoughts on this?
- Is there any type of support/help you would have liked that you did not receive?
- Tell us about your experiences of this/these different interventions/? Independent vs. guided interventions? Helpful/not helpful? In what way? What made you participate/abstain?)
